# An evaluation of psychometric properties of caregiver burden outcome measures used in caregivers of children with cerebral palsy: a systematic review protocol

**DOI:** 10.1186/s13643-016-0219-3

**Published:** 2016-03-09

**Authors:** Jermaine M. Dambi, Jennifer Jelsma, Tecla Mlambo, Matthew Chiwaridzo, Nyaradzai Dangarembizi-Munambah, Lieselotte Corten

**Affiliations:** University of Cape Town, Cape Town, South Africa; Department of Rehabilitation, College of Health Sciences, University of Zimbabwe, P O Box AV 178, Avondale, Harare, Zimbabwe; Division of Physiotherapy, Department of Health and Rehabilitation Sciences, Faculty of Health Sciences, University of Cape Town, Anzio Road, Observatory, Cape Town, South Africa

**Keywords:** Cerebral Palsy, Caregiver burden/strain, Outcome measurement, Psychometric properties, Evaluation, Systematic review, Protocol

## Abstract

**Background:**

Cerebral palsy (CP) is the most common, life-long paediatric disability. Taking care of a child with CP often results in caregiver burden/strain in the long run. As caregivers play an essential role in the rehabilitation of these children, it is therefore important to routinely screen for health outcomes in informal caregivers. Consequently, a plethora of caregiver burden outcome measures have been developed; however, there is a dearth of evidence of the most psychometrically sound tools. Therefore, the broad objective of this systematic review is to evaluate the psychometrical properties and clinical utility of tools used to measure caregiver burden in caregivers of children with CP.

**Methods/design:**

This is a systematic review for the evaluation of the psychometric properties of caregiver burden outcome tools. Two independent and blinded reviewers will search articles on PubMed, Scopus, Web of Science, CINAHL, PsychINFO and Africa-Wide Google Scholar. Information will be analysed using predefined criteria. Thereafter, three independent reviewers will then screen the retrieved articles. The methodological quality of studies on the development and validation of the identified tools will be evaluated using the four point COnsensus-based Standards for the selection of health Measurement INstruments (COSMIN) checklist. Finally, the psychometric properties of the tools which were developed and validated from methodological sound studies will then be analysed using predefined criteria.

**Discussion:**

The proposed systematic review will give an extensive review of the psychometrical properties of tools used to measure caregiver burden in caregivers of children with CP. We hope to identify tools that can be used to accurately screen for caregiver burden both in clinical setting and for research purposes.

**Systematic review registration:**

PROSPERO CRD42015028026.

## Introduction

Provision of care for a child with a long-term health condition is often associated with negative health outcomes in caregivers, for instance, depression, stress, anxiety and low self-efficacy were reported in caregivers [1–6]. Cerebral palsy (CP) is the most common paediatric disability causing long-term functional limitations [7, 8]. Children with CP most often present with multiple impairments, activity limitations and participation restrictions [9, 10]. More so, due to its diverse and complex presentation, CP is envisaged as the prototype paediatric disability [7, 8]. As such, most children require lifetime extensive assistance in functional day to day activities [10–12]. The level of required assistance depends on the severity of impairments, activity limitations and participation restrictions [13]. Taking care of a child is part of normal parenthood; however, the excessive demands associated with taking care of a child with a disability may lead to increased burden/strain [5, 14]. Consequently, long-term caregiving for a child with CP may negatively affect the well-being of caregivers [4, 12, 15].

Caregiver burden has been defined as “strain or load borne by a person who cares for a family member with a disability” [16]. Caregiver burden is multifactorial complex, subjective and dynamic as envisaged in different conceptual models which have been developed to explain this construct [16–19]. The conceptual model by Raina et al. (2004) is one of the mostly cited and applied caregiver burden conceptual frameworks [19]. It postulates that caregiver burden is an interaction between the caregivers’ background, contextual factors, child characteristics, intrapsychic factors and coping factors [19]. For instance, presence of behavioural problems in children with CP, caregivers’ socio-economic status, availability of social support and caregivers’ self-efficacy all affect the overall perception of the burden of care [19]. Although usage of diverse semantics in describing caregiver burden makes it difficult to come up with a universally conceptualized definition and model, it is clear that long-term caregiving may lead to physical, psychological, emotional, social and financial strain [16–18].

With the advent of patient-centred and family-centred approaches to clinical care, the need to evaluate services from the perspective of patients, specifically the perceived impact of care on patients’ well-being, becomes more inherent [20]. More so, it is essential to evaluate the health outcomes in caregivers as they are an invaluable resource in the rehabilitation of children with long-term disabilities [5, 6]. For instance, the caregiver acts as the provider, decision maker, companion, custodian and advocator for a child with a disability [21]. Thus, routine assessment/screening of caregiver burden is of paramount importance for optimal functional outcomes of children with disabilities.

Given the well-documented effects of caregiving on the health of caregivers, it is important to routinely screen for caregivers burden/strain [5]. This is only attainable through the use of psychometrically sound outcome measures [14]. According to the COnsensus-based Standards for the selection of health Measurement INstruments (COSMIN) guidelines, an outcome measure has to be valid, reliable and responsive in order for it to adequately capture the construct it is purposed to measure [22]. Over the past few decades, there has been an exponential increase in the number of outcome measures to evaluate burden of care [14]. However, some of them are generic in nature and their utility in measuring burden of care in caregivers of children with disabilities may be questionable [14, 23, 24]. Further, there is a paucity of systematic evidence of the psychometric rigour of tools that have been used to measure the caregivers’ burden while taking care of a child with a disability.

Therefore, the broad objective of this systematic review is to evaluate the psychometrical properties of tools that have been utilized to measure caregiver burden in caregivers of children with CP. The specific objectives are to:

1. Identify tools used to measure perceived caregiver burden in caregivers of children with CP
2. Evaluate the psychometric properties of the identified outcome measurement tools
3. To evaluate the clinical utility of the identified outcome measurement tools

## Methods

### Protocol/ registration

In conducting this review, we will utilize the Preferred Reporting Items of Systematic Reviews and Meta-Analyses Protocol (PRISMA-P) guidelines [25]. The protocol has been registered on PROSPERO database (Ref: CRD42015028026).

### Eligibility criteria

In selecting the studies, we will apply the following criterion:

#### Study designs/interventions

We will give precedence to articles on the development and validation of tools for measuring caregiver burden in caregivers of children with CP and/or other physical disabilities such as spinal bifida hydrocephalus among others. In as much as CP is considered to be a prototype paediatric disability [7, 8], we hypothesize that the burden of caring may be equivalent, regardless of the causative condition .Therefore, we will also include other disabilities such as hydrocephalus, spinal bifida among others. We will also consider studies that evaluated caregiver burden/strain in informal caregivers of children with CP and/or other disabilities or interventional studies with caregiver burden as an outcome measurement. We will exclude systematic reviews, qualitative studies, case studies and editorial letters. To capture as much information as possible, all quantitative study designs will be considered.

#### Participants

We will include studies examining the perceived burden of care in informal caregivers (18 years or older) of children with disabilities in the age range 0–12 years. We are cognisant of the changes in the dynamics of caregiving along the developmental trajectory. For instance, the dynamics of caregiving for a teenager with a disability may be different from providing care for a child in the age range 0–12 years [26]. Additionally, in the present review, an informal caregiver is defined as someone who takes the primary responsibility for caregiving a child with CP and are not formally educated nor remunerated for assuming the caregiver role.

#### Language

We will be selecting articles published in English language only as we do not have the financial resources for the translation of articles published in other languages. Further, when we performed our preliminary searches, we did not come across tools that were developed in languages other than English.

#### Information sources

We will search the following databases from their inception up to September 2015: PubMed, CINAHL, Scopus, PsychINFO and Africa-Wide information. To ensure literature saturation, where only abstracts are available online, we will first try to contact the authors to see if a full text is available. If, after consulting the authors, only an abstract is available, we will exclude this abstract from our review.

We will also review grey literature, i.e. we will use the Google Scholar search engine to search potential databases such as university databases conference proceedings among others for articles. For completeness, the reference lists of identified articles will also be manually searched.

#### Search strategy

Outlined in Table 1 below is an example of how we will search for the articles in CINAHL database.

**Table 1:** Search strategy

Key word	Alternative words
Caregiver	Carer* OR mother OR parent* OR legal guardian*
Children	Child* OR paediatric* OR toddler* OR infants* OR pediatric*
Cerebral palsy	CP OR physical disability OR disability* OR neurodev* disorder*OR traumatic brain injur*
Burden	Strain OR stress OR burnout
Outcome measure	Tool OR questionnaire OR scale OR assessment
Psychometric	Validity OR reliability OR responsiveness
evaluation	Determination OR measurement
Development	Construction

As an illustration, we will input the following key words to search articles in the CINAHL database: (“Caregiver” OR “care*” OR “mother”) AND (“burden” OR “strain” OR “stress” OR “distress”) AND (“outcome” OR “tool” OR “scale”) AND( “valid*” OR “reliability” AND “dev*”) AND (“CP” OR “cerebral palsy” OR “disabilit*” OR “long term health condition”) AND (“child” OR “paediatr*” AND “development” OR “construction”).

#### Data management

The articles will be imported into RevMan (version 5.3) data management software. The electronic searches will also be saved on users’ PubMed Scopus and EBSCOhost accounts. We will also print the summaries of all the searches to enhance the data capturing of the search records. The principal investigator will create a Google Drive folder which will act as repository of all the articles, which will be made available to all authors.

#### Data collection process

The principal author (JD) will search the databases and extract the titles and abstracts for further investigation. Thereafter, two researchers (NM and MC) will independently retrieve the full manuscripts of articles deemed relevant. Two other independent reviewers (LC and TM) will blindly screen the retrieved articles using a standardized data collection form. Information to be extracted will include the research setting and design, study sample, demographics of the participants, mode of administration, number of items, cost, total possible score and the year in which the tool was developed. In the event of a disagreement, a third reviewer (JJ) will make the final decision.

#### Outcomes and prioritization

For this review caregiver burden/strain will be the primary outcome measure.

#### Risk of bias individual studies

The four-point COSMIN checklist [22, 27] will be used to assess the methodological quality of the reviewed studies. This is essential to prevent the risk of selecting and evaluating tools which were developed using designs with poor methodological rigour [22]. The COSMIN checklist rates the rigour of the reliability, validity, responsiveness, hypothesis testing, interpretability and generalizability of studies on the development and use of health-related patient-reported outcomes [22, 27]. Items are rated on a four-point Likert scale, i.e. “excellent”, “good”, “fair” and “poor”. Where details are not published, the authors of the article will be contacted to achieve the most truthful rating of the assessment tool and to decrease bias in the analysis.

#### Psychometric properties and data extraction

The psychometrical properties will be evaluated using the checklist as outlined by Terwee et al. [28] (See Table 2). Each psychometric property can be rated as positive negative or questionable. An ideal tool should possess positive ratings [28].

**Table 2:** Quality criterion for psychometrical properties [28]

Property	Definition	Quality criteria
1. Content validity	The extent to which the domain of interest is comprehensively sampled by the items in the questionnaire	+ A clear description is provided of the measurement aim, the target population, the concepts that are being measured, and the item selection AND target population and (investigators OR experts) were involved in item selection; ? A clear description of above-mentioned aspects is lacking OR only target population involved OR doubtful design or method; − No target population involvement; 0 No information found on target population involvement.
2. Internal consistency	The extent to which items in a (sub)scale are intercorrelated, thus measuring the same construct	+ Factor analyses performed on adequate sample size (7 * # items and >100) AND Cronbach’s alpha(s) calculated per dimension AND Cronbach’s alpha(s) between 0.70 and 0.95; ? No factor analysis OR doubtful design or method; − Cronbach’s alpha(s) <0.70 or >0.95, despite adequate design and method; 0 No information found on internal consistency.
3. Criterion validity	The extent to which scores on a particular questionnaire relate to a gold standard	+ Convincing arguments that gold standard is “gold” AND correlation with gold standard >0.70; ? No convincing arguments that gold standard is “gold” OR doubtful design or method; − Correlation with gold standard <0.70, despite adequate design and method; 0 No information found on criterion validity.
4. Construct validity	The extent to which scores on a particular questionnaire relate to other measures in a manner that is consistent with theoretically derived hypotheses concerning the concepts that are being measured	+ Specific hypotheses were formulated AND at least 75 % of the results are in accordance with these hypotheses; ? Doubtful design or method (e.g. no hypotheses); − Less than 75 % of hypotheses were confirmed, despite adequate design and methods; 0 No information found on construct validity.
5. Reproducibility 5.1. Agreement	The extent to which the scores on repeated measures are close to each other (absolute measurement error)	+ MIC < SDC OR MIC outside the LOA OR convincing arguments that agreement is acceptable; ? Doubtful design or method OR (MIC not defined AND no convincing arguments that agreement is acceptable); − MIC > SDC OR MIC equals or inside LOA, despite adequate design and method; 0 No information found on agreement.
5.2. Reliability	The extent to which patients can be distinguished from each other, despite measurement errors (relative measurement error)	+ ICC or weighted Kappa >0.70; ? Doubtful design or method (e.g., time interval not mentioned); − ICC or weighted Kappa <0.70, despite adequate design and method; 0 No information found on reliability.
6. Responsiveness	The ability of a questionnaire to detect clinically important changes over time	+ SDC or SDC < MIC OR MIC outside the LOA OR RR >1.96 OR AUC >0.70; ? Doubtful design or method; − SDC or SDC > MIC OR MIC equals or inside LOA OR RR <1.96 OR AUC <0.70, despite adequate design and methods; 0 No information found on responsiveness. 0 No information found on responsiveness.
7. Floor and ceiling effects	The number of respondents who achieved the lowest or highest possible score	+ <15 % of the respondents achieved the highest or lowest possible scores; ? Doubtful design or method; − >15 % of the respondents achieved the highest or lowest possible scores, despite adequate design and methods; 0 No information found on interpretation.
8. Interpretability	The degree to which one can assign qualitative meaning to quantitative scores	+ Mean and SD scores presented of at least four relevant subgroups of patients and MIC defined; ? Doubtful design or method OR less than four subgroups OR no MIC defined; 0 No information found on interpretation.

*MIC* minimal important change, *SDC* smallest detectable change, *LOA* limits of agreement, *ICC* Intraclass correlation, *SD* standard deviation

Symbols: + positive rating; ? indeterminate rating (doubtful design or method—lacking of a clear description of the design or methods of the study, sample size smaller than 50 subjects (should be at least 50 in every (subgroup) analysis), or any important methodological weakness in the design or execution of the study); − negative rating; 0 no information available

#### Best evidence synthesis

Where a tool has validated in several studies, we will combine the findings to come with the best evidence for that particular tool. We will use a previously established criterion for synthesizing evidence by the Cochrane Collaboration Back Review Group [29] as outlined in Table 3.

**Table 3:** Levels of evidence for the overall quality of the measurement property [29]

Level	Rating	Criteria
Strong	+++ or − −	Consistent findings in multiple studies of good methodological quality OR in one study of excellent methodological quality
Moderate		Consistent findings in multiple studies of fair methodological quality OR in one study of good methodological quality
Limited	+ or −	One study of fair methodological quality
Conflicting	+/−	Conflicting findings
Unknown	?	Only studies of poor methodological quality

+ positive result, − negative result

## Discussion

We hope to identify the most psychometrically sound, caregiver burden outcome measures. This is important given a plethora of tools which have been developed to measure this multidimensional construct. This is especially important for clinical use as there is a great need to routinely screen of caregivers at risk or who are exhibiting signs of strain/distress. Identification and use of caregiver burden outcomes with rigorous psychometrical properties will also enhance the credibility, methodological rigour and overall external validity and comparability of interventions for improving caregiver burden.

## Abbreviations

CINAHL: Cumulative Index of Nursing and Allied Health Literature
COSMIN: COnsensus-based Standards for the selection of health Measurement Instruments
CP: cerebral palsy
PRISMA-P: Preferred Reporting Items of Systematic Reviews and Meta-Analyses Protocol
